# The influence of the operator's experience on the microleakage of two universal adhesives

**DOI:** 10.1002/cre2.458

**Published:** 2021-05-26

**Authors:** Fereshteh Shafiei, Zahra Dehghani, Zahra Jowkar

**Affiliations:** ^1^ Oral and Dental Disease Research Center, Department of Operative Dentistry, School of Dentistry Shiraz University of Medical Sciences Shiraz Iran

**Keywords:** microleakage, operator's experience, universal adhesive

## Abstract

**Objectives:**

This study aimed to investigate the influence of the operator's experience on the microleakage of two universal adhesives applied in self‐etch (SE) and etch‐and‐rinse (E&R) modes.

**Materials and methods:**

Two class V cavities were prepared on the buccal and lingual surfaces of 112 caries‐free extracted human molars and were divided into eight groups based on the combination of the factors “adhesive system” (Tetric N‐Bond Universal Adhesive [TNU]; Scotchbond Universal Adhesive [SBU]), “adhesive strategy” (SE or E&R), and “the operators performing the procedures” (specialists in operative dentistry or intern students). After 24 h of water storage and thermocycling, the marginal microleakage was evaluated using the dye penetration technique and the data were analyzed.

**Results:**

In the SE mode, microleakage at the enamel margin for both adhesives was higher in the student group than in the specialist group (*p* values < 0.05). The operator's skill did not affect the microleakage scores of both adhesives applied in the E&R mode at the dentin and enamel margins or in the SE mode at the dentin margins (*p* > 0.05). The microleakage score was significantly higher at the dentin margins than the enamel margins for both universal adhesives applied in the E&R mode (*p* values <0.05).

**Conclusion:**

The microleakage values of the universal adhesives applied in the SE mode at the enamel margins were affected by the operator's skill. However, the operator's experience did not affect the microleakage scores of the two universal adhesives applied in the E&R mode at the enamel and dentin margins or in the SE mode at the dentin margins.

## 1 INTRODUCTION

One of the most important influential factors on the long‐term performance of composite restorations is providing a flawless seal resistant to contamination from oral fluids or the absence of leakage at the material/tooth interface (Zanatta et al., 2017). Microleakage remains an important cause of composite restoration failure despite the improvements in restorative materials (Zanatta et al., 2017). Several factors such as material components, physical characteristics of the composite resin, polymerization methods, and cavity preparation design parameters may lead to microleakage at the tooth‐restoration interface (Kalmowicz et al., 2015; Schneider et al., 2010). Moreover, clinical variables (such as material manipulation and application technique), isolation constraints, and adequate knowledge of composite resins and adhesive science may affect the marginal integrity of composite restorations (Karaman et al., 2013).

The polymerization shrinkage of the composite can lead to micro‐gap formation, microleakage, debonding of the material from the tooth structure, recurrent caries, marginal staining, sensitivity, possible pulpal inflammation, and partial or complete restoration replacement (Sahebi et al., 2012; Schneider et al., 2010). Therefore, some efforts have been made to enhance the adhesion between dental substrates and restorative materials for achieving durable and leakage‐free restorations (Karaman et al., 2013).

Universal adhesives are the latest generation of adhesives which may be applied in 2‐step etch‐and‐rinse (E&R) or 1‐step self‐etch (SE) modes depending on the clinician's preference (Isolan et al., 2014; Shafiei et al., 2019). Promising results have recently been presented for universal adhesives regarding the degree of conversion inside the hybrid layer, nanoleakage, bond strength, and the long‐term effectiveness of dentin bonding (de Oliveira da Rosa et al., 2015).

When universal adhesives are applied in E&R mode, the first step is etching with phosphoric acid. This step may lead to the increased technique sensitivity of applying the adhesive because a discrepancy may occur between the depth of dentin demineralization and hybridization (Vaidyanathan & Vaidyanathan, 2009).

Universal adhesives can also be applied in SE mode. One of the advantages of the SE approach is that dentin demineralization and priming occur simultaneously (Sezinando et al., 2015). Moreover, it has been proposed that the fewer application steps of the SE approach reduce technique sensitivity and the application time of the SE adhesives (Van Meerbeek et al., 2011). However, some variables related to the application of SE adhesive systems should be controlled by the clinician including a certain amount of moisture necessary for dentin bonding, proper timing for adhesive application, the air‐drying step, and the required rubbing action during application (Van Meerbeek et al., 2011).

Generally, multiple steps involved in bonding procedures are prone to different errors. Hence, it has been proposed that the success of the adhesive system not only is dependent on material‐related factors but also is affected by the operator's performance and skill (Ueda et al., 2010). It has been demonstrated that the operator's experience can affect the efficacy of adhesive systems in bonding to tooth structures (Ueda et al., 2010).

Based on the authors' knowledge, there are no published data about the effect of the operator's experience on the microleakage of universal adhesives. Therefore, this study aimed to evaluate the effect of the operator's experience on the microleakage of the universal adhesive systems used in the E&R and SE modes.

## 2 MATERIALS AND METHODS

112 fresh caries‐free human molars with similar sizes and anatomic shapes extracted for orthodontic reasons were collected for this in vitro study with the written consent of each patient. After being examined under a stereoscopic microscope (Carl Zeiss, Oberkochen, Germany) for the absence of crack, fracture, abrasion, previous restorations, or structural deformities, the teeth were cleaned with a periodontal curette and stored at 4°C in a 0.5% chloramine T solution (Fisher Chemical, Fair Lawn, NJ, USA) to prevent bacterial growth. They were used within 6 months. The study protocol was approved by the Research and Ethics Committee of Shiraz University of Medical Sciences (Protocol # IR.SUMS.DENTAL.REC.1399.052).

Class V cavities (2.0 mm depth, 3.0 mm height, and 3.0 mm width) with occlusal cavosurface margins located 1.5 mm coronal from the cemento‐enamel junction (CEJ) and gingival cavosurface margins located 1.5 mm below the CEJ were prepared without bevels in the buccal and lingual surfaces of each tooth using fissure diamond burs (Diamond Fissure 330; SS White) in a water‐cooled high‐speed handpiece. The bur was changed after every five preparations. The cavity sizes were checked by a periodontal probe (PCP UNC 127, Hu‐Friedy Mfg. Co. Inc., Chicago, IL) after the tooth preparations. All the cavities were prepared by the same calibrated operator. After the completion of the preparations, the cavities were cleaned with pumice paste, rinsed with a water spray, and gently air‐dried.

Two universal adhesive systems (used with either the SE or the E&R approach) were tested in this study including Tetric N‐Bond Universal Adhesive (TNU, Ivoclar Vivadent, Liechtenstein) and Scotchbond Universal Adhesive (SBU, 3 M ESPE, St Paul, MN, USA). The teeth were then randomly divided into two main groups (*n* = 56). The bonding procedures and restorations of the cavities were performed by 8 specialists with 10–12 years of professional clinical experience in one group (*n* = 56) and by 8 last‐year dental students at Shiraz School of Dentistry in another group (*n* = 56). A closed envelope containing the instructions about what type of adhesive to use for each cavity was given to each operator. Each main group was randomly divided into four subgroups (1–4) of 14 cavities each. In subgroup 1, TNU was applied in the E&R mode, and in subgroup 2, TNU was applied in the SE mode. In subgroup 3, SBU was applied in the E&R mode, and in subgroup 4, SBU was applied in the SE mode. All adhesives were applied according to the manufacturer's instructions.

All the cavities were restored with composite Z250 (3M ESPE, St Paul, MN, USA) using an incremental technique after application and light activation of its respective adhesive bonding system according to the manufacturer's instructions. Each increment was cured for 40 seconds using a light‐curing unit (VIP Junior, Bisco, Schaumburg, IL) at 600 mW/cm^2^.

**FIGURE 1:**
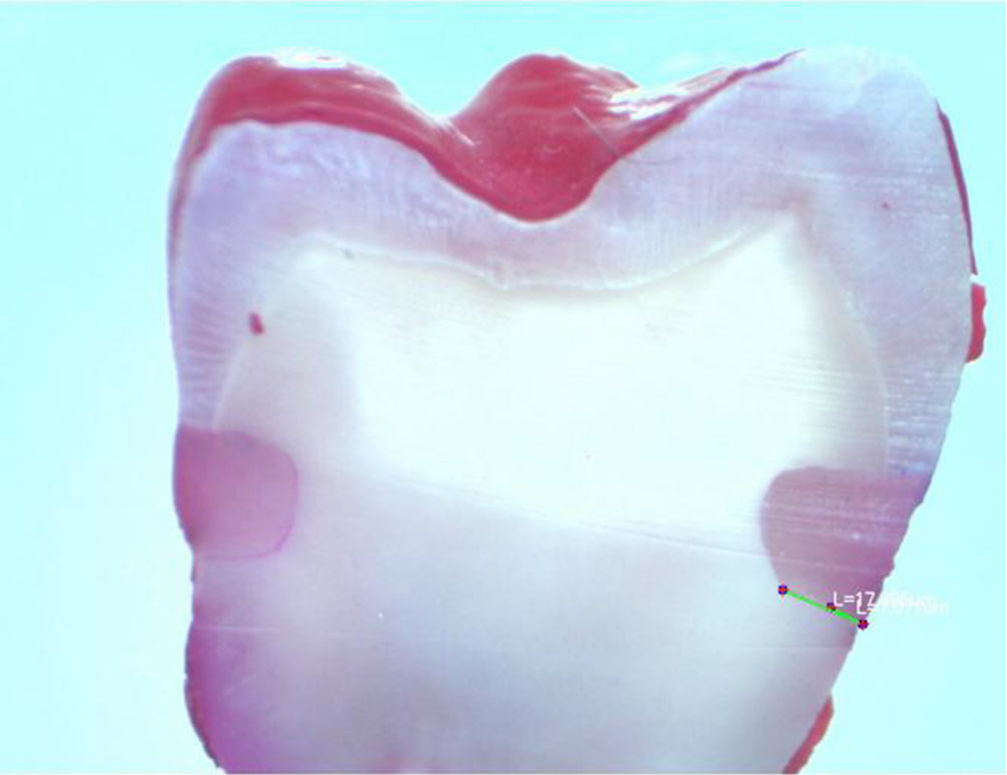
Longitudinal section of molar showing dye penetration through the interface between the composite resin and the tooth structure

The teeth were thermo‐cycled for 1500 cycles between 5 and 55°C with a dwell time of 10 s in each water bath. After sealing the root apices of the teeth with utility wax, all the areas of the teeth except for the restorations and a 1‐mm rim of the tooth structure around each restoration were covered with two coats of nail varnish. Subsequently, the specimens were immersed in 0.5% basic fuchsin dye solution for 24 h at room temperature. Then, they were rinsed thoroughly under tap water for 1 h and sectioned buccolingually in the middle of the restorations using a water‐cooled low‐speed cutting machine (Mecatome T201 A, Presi, Grenoble, France) to obtain two sections for each restoration. Each section was blindly examined for dye penetration by a calibrated operator under a digital microscope (Dino Lite, Taipei, Taiwan). Linear dye penetration was measured and recorded separately along the occlusal and gingival walls of the restorations as a percentage of the total length of the gingival or occlusal wall (Figure 1). All measurements were performed using Dino‐Lite Pro software.

The obtained results were statistically analyzed using the Kruskal–Wallis test to compare the eight groups and the Mann–Whitney test for paired comparisons. The occlusal and gingival margins of the groups were compared using the Wilcoxon rank‐sum test (*p* < 0.05).

## 3 RESULTS

The mean microleakage percentages and SDs at the enamel and dentin margins of class V composite restorations are presented in Tables 1 and 2. For both adhesives applied in the SE mode, microleakage at the enamel margin was higher in the student group than in the specialist group (*p* < 0.05). When applied in the SE mode, the microleakage scores of both adhesives were not affected by the operator's skill at the dentin margins (*p* > 0.05). The operator's skill did not affect the microleakage scores of both adhesives applied in the E&R mode at the dentin and enamel margins (*p* > 0.05).

**TABLE 1:** Mean microleakage percentage (SD) of the experimental groups at the enamel and dentin margins in the specialist groups

Experimental groups	Enamel margin microleakage	Dentin margin microleakage
	Mean (±SD)	Mean (±SD)
Scotchbond universal adhesive (SE)	1.6 (±3.05)^Aa^	3 (±5.2)^Aa^
Scotchbond universal adhesive (E&R)	1.1 (±2.3)^Aa^	9.3 (±10.01)^Ab^
Tetric N‐Bond universal adhesive(SE)	1.3 (±3.6)^Aa^	2.6 (±5.4)^Aa^
Tetric N‐Bond universal adhesive(E&R)	1 (±1.9)^Aa^	7.6 (±9.6)^Ab^

*Note*: Within each row, mean values with different lowercase superscript letters show statistically significant differences at a significance level of 0.05. Within each column, mean values with different uppercase superscript letters show statistically significant differences at a significance level of 0.05.

Abbreviations: E&R, etch and rinse; SE, self‐etch.

**TABLE 2:** Mean microleakage percentage (SD) of the experimental groups at the enamel and dentin margins in the student groups

Experimental groups	Enamel margin microleakage	Dentin margin microleakage
	Mean (±SD)	Mean (±SD)
Scotchbond universal adhesive (SE)	7.6 (±8.2)^Aa^	4.3 (±6.8)^Aa^
Scotchbond universal adhesive (E&R)	2.06 (±3.2)^Aa^	7.2 (±8.7)^Ab^
Tetric N‐Bond universal adhesive (SE)	8.8 (±9.1)^Aa^	2.5 (±6.06)^Ab^
Tetric N‐Bond universal adhesive (E&R)	1.4 (±3.2)^Ba^	8.0 (±9.7)^Ab^

*Note*: Within each row, mean values with different lowercase superscript letters show statistically significant differences at a significance level of 0.05. Within each column, mean values with different uppercase superscript letters show statistically significant differences at a significance level of 0.05.

Abbreviations: E&R, etch and rinse; SE, self‐etch.

According to the Kruskal–Wallis test, there were significant differences between the microleakage scores of the two universal adhesives at the enamel margins in the student group (*p* = 0.03). At the enamel margins, the highest microleakage score was observed in TNU applied in the SE mode, followed by SBU applied in the SE mode, and then SBU applied in the E&R mode in the student group, whereas the lowest microleakage score was reported in TNU applied in the E&R mode in the student group. In the specialist group, no significant differences were found among the microleakage scores of the two universal adhesives at the enamel or dentin margins (*p* > 0.05).

Comparing microleakage at the enamel and dentin margins in the specialist group using the Wilcoxon signed ranks test analysis showed that the microleakage score was significantly higher at the dentin margins than the enamel margins for both universal adhesives applied in the E&R mode (*p* < 0.05). When SBU or TNU was applied in the SE mode in the specialist group, no significant differences were found between the microleakage scores at the enamel and dentin margins (*p* > 0.05). When TNU was applied in the SE mode in the student group, the microleakage score was significantly higher at the enamel margins than the dentin margins (*p* < 0.05). When TNU or SBU was applied in the E&R mode in the student group, the microleakage score was significantly higher at the dentin margins than the enamel margins (*p* < 0.05).

## 4 DISCUSSION

The present study evaluated the effect of the operator's experience on the microleakage of two universal adhesive systems (TNU and SBU) applied in the E&R or SE mode. According to the results of the present study, the operator's skill did not affect the microleakage scores of the SBU and TNU adhesives applied in the E&R mode at the dentin and enamel margins. When the two adhesives were applied in the SE mode, the microleakage score at the enamel margins was higher in the student group than in the specialist group. At the dentin margins, the microleakage scores of both adhesives applied in the SE mode were not affected by the operator's skill.

In this study, the microleakage of the two universal adhesives was measured by the dye penetration technique. The in vitro dye penetration technique remains a valuable preclinical screening test for comparing the sealing ability of different adhesive materials and techniques (Alnakib & Alsaady, 2021; Shafiei et al., 2017; Shafiei et al., 2018).

To obtain the microleakage percentage in the present study, after measuring the occlusal and gingival microleakage in Mm digitally by computer software, they were divided by the total length of the gingival or occlusal wall and multiplying by 100%. This technique scores dye penetration numerically. This technique is more objective and precise with less chance of bias compared to the scoring method which relies on the subjective evaluation of the observer (Alnakib & Alsaady, 2021).

This study showed that the operator's skill did not affect the microleakage scores of the two universal adhesives applied in the SE or E&R mode at the dentin margins. This finding supports the potential of the studied universal adhesives to be used by each operator as multimodal adhesives at the dentin margins.

One of the universal adhesives used in the present study was SBU. SBU can form a chemical bond to dentin which is associated with the interaction of the polyalkenoic acid co‐polymer (also known as Vitrebond copolymer) with calcium in hydroxyapatite, forming stable calcium‐MDP complexes within the partially demineralized dentin through nano‐layering (Yoshihara et al., 2013). It has been reported that the chemical bond between the adhesive and calcium in hydroxyapatite creates a stable interface even without micromechanical retention (Costa et al., 2018). Moreover, SBU is a hydroxyethyl methacrylate (HEMA)‐containing adhesive. HEMA is added to SBU to enhance dentin wettability, monomer infiltration, and water sorption after polymerization (Costa et al., 2018; Takahashi et al., 2011; Van Meerbeek et al., 2003). Another important purpose of the incorporation of HEMA into adhesives is to prevent hydrophobic monomer/water phase separation (Takahashi et al., 2011; Van Landuyt et al., 2005).

Another universal adhesive which was used in the present study was TNU. TNU is a mild etching adhesive which contains a combination of hydrophilic (HEMA), hydrophobic (decandiol dimethacrylate [D3MA]), and intermediate (bis‐GMA) monomers. Thus, it can effectively bond to the dentin and composite resin and bridge the gap between them (Jayasheel et al., 2017).

It should be noted that the two universal adhesives used in the present study are ethanol‐ and water‐based adhesives. Ethanol is an organic solvent that acts as a carrier and water chaser. Thus, it allows the dentin surface to remain moist for bonding and facilitates the penetration of resin monomers into moist dentin (Mobarak et al., 2013). Hence, the marginal seal in the dentin may be less affected by the practitioner's clinical skill in these ethanol‐based adhesives. The statistically similar values for the microleakage of the two universal adhesives at the dentin margins in the specialist and student groups could be attributed to the water and ethanol content of the two universal adhesives, possibly reducing the technique sensitivity of applying the adhesives.

In the present study, the operator's skill did not affect the microleakage scores of the two universal adhesives applied in the E&R mode at the enamel or dentin margins. These results may be justified by the bonding mechanisms of the two universal adhesives to enamel. SBU (pH = 2.7) is considered as an ultra‐mild to mild acidic adhesive system (McLean et al., 2015). TNU is also a mild‐etching adhesive with a pH of approximately 2.5–3.0 (Jayasheel et al., 2017). Therefore, acid etching can improve the mechanical interlocking of the two universal adhesives applied to the tooth structure in the E&R mode (McLean et al., 2015).

Based on the results of the present study, the microleakage scores of both adhesives applied in the SE mode were sensitive to the operator's skill at the enamel margins and were higher in the student group than in the specialist group. This finding might be related to the insufficient experience of the students with SE adhesives. Poor adhesion and marginal discrepancies at the enamel margins in the SE mode in the student group may also be attributed to the fact that SE adhesives do not etch enamel as much as phosphoric acid does (Erickson et al., 2006; McLean et al., 2015). Therefore, it seems that universal adhesives applied at enamel margins in the SE mode might be more sensitive to the operator's skill compared to when they are applied in the E&R mode due to the lower bond strength of SE adhesives to enamel than that of E&R adhesives (McLean et al., 2015).

Both universal adhesives used in the present study contain 10‐methacryloyloxydecyl dihydrogen phosphate (10‐MDP) which possesses the capacity to chemically interact with hydroxyapatite. The low bonding effectiveness of mild self‐etch adhesives to enamel may partly be related to the low chemical reactivity of 10‐MDP with enamel hydroxyapatite because the etching, primer, and adhesive components are all in one bottle in the universal adhesives applied in the SE mode (McLean et al., 2015).

According to the results of the present study, when the two universal adhesives were applied in the SE mode in the student group, higher microleakage scores were recorded compared to when they were used in the E&R mode at the enamel margins. This result is in line with a previous study which demonstrated that etching the enamel significantly increased the bond strength of universal adhesives to enamel (McLean et al., 2015). It seems that the additional acid etching step to remove the smear layer may have improved the infiltration of the two universal adhesive systems into the hybrid layer. This may potentially have improved the micromechanical bonds between the highly mineralized enamel substrate and the composite resin (McLean et al., 2015).

The present study also showed that in the specialist group, no significant differences were found among the microleakage scores of the two universal adhesives at the enamel or dentin margins. This might be attributed to the operator's skill and special attention during the application of the adhesives in the specialist group. Therefore, specialists can use universal adhesives in either the E&R mode or the SE mode without compromising the microleakage scores at the enamel or dentin margins.

The results of the present study also showed that the microleakage score in the specialist group was significantly higher at the dentin margins than the enamel margins for both universal adhesives applied in the E&R mode. This finding may be attributed to the excessive drying of the dentin after acid etching application which may lead to the collapse of collagen fibers and reduce the bonding performance. On the other hand, excessive moisture on the dentin may potentially lead to the separation of the hydrophobic and hydrophilic adhesive components, forming a gap at the dentin‐tooth interface (Park et al., 2009). Unlike the SBU, the microleakage score was significantly higher at the enamel margin than the dentin margin for the TNU applied in the SE mode in the student group. The differences in the compositions of the two adhesives could be an explanation for this result.

When applied in the SE mode, the microleakage scores of the used adhesives at the dentin margin were not affected by the operator's skill in the present study. However, at the enamel margins, both universal adhesives applied in the SE mode showed higher microleakage values in the student group than in the specialist group. Therefore, it seems that the students showed better results when using universal adhesives in the E&R mode compared to the SE mode at the enamel margins. To achieve better results, selective etching of enamel margins is recommended for the students when using universal adhesives. However, sufficient knowledge of the application procedure for each adhesive and the operator's experience are necessary to achieve a tight seal for the clinical success of composite restorations (Unlu et al., 2012).

Several factors may lead to microleakage at the tooth‐restoration interface such as material components, physical characteristics of the composite resin, polymerization methods, and cavity preparation design parameters (Kalmowicz et al., 2015; Schneider et al., 2010). Moreover, clinical variables (such as material manipulation and application technique), isolation constraints, and adequate knowledge of composite resins and adhesive science may affect the marginal integrity of composite restorations (Karaman et al., 2013). The polymerization shrinkage of the composite can also lead to micro‐gap formation and microleakage (Schneider et al., 2010). In the present study, the effects of the operator's skill and mode of application of the universal adhesives on the microleakage of composite resin restorations were assessed. Future studies regarding other causes of microleakage are needed to explore the efficacy of universal adhesives used by operators with different levels of experience in clinical practice.

This study had some limitations. In the present study, microleakage of the universal adhesives was evaluated and further studies evaluating other important criteria on the operator's experience such as bond strength to enamel and dentin should be performed. It should also be noted that the significant factors identified in standardized laboratory studies may not directly predict the results of a clinical multi‐operator trial. Further studies dealing with different adhesive systems, more operator‐dependent variables, and different operator‐material combinations are necessary to identify and characterize the interactions between the operator and the materials.

## 5 CONCLUSION

The microleakage values of the TNU and SBU were comparable to each other regardless of the application mode. When the two universal adhesives were applied in the self‐etch mode, microleakage at the enamel margin was higher in the student group than in the specialist group. Therefore, it seems that universal adhesives applied in the SE mode at enamel margins are more sensitive to the operator's skill compared to when they are applied in the E&R mode. However, the operator's skill did not affect the microleakage scores of the two universal adhesives when they were applied in the E&R mode at the enamel and dentin margins or in the self‐etch mode at the dentin margins.

## CONFLICT OF INTEREST

The authors do not have any conflicts of interest to declare.

## AUTHOR CONTRIBUTIONS

Conceptualization; data curation; formal analysis; investigation; methodology; software; visualization; writing‐original draft; writing‐review and editing: Fereshteh Shafiei. Data curation; formal analysis; investigation; methodology; software; visualization; writing‐original draft: Zahra Dehghani. Data curation; formal analysis; investigation; methodology; software; visualization; writing‐original draft; writing‐review and editing: Zahra Jowkar.

## ETHICS STATEMENT

The study protocol was approved by the Research and Ethics Committee of Shiraz University of Medical Sciences (Protocol # IR.SUMS.DENTAL.REC.1399.052) and was conducted in compliance with the Declaration of Helsinki.

## Data Availability

The data that support the findings of this study are available on request from the corresponding author.
